# Perceiving gloss through transparency

**DOI:** 10.1177/20416695251355381

**Published:** 2025-07-22

**Authors:** Sabrina Hansmann-Roth, Pascal Mamassian

**Affiliations:** 1 Icelandic Vision Lab, Department of Psychology, 63541 University of Iceland, Reykjavík, Iceland; 2 Laboratoire des systèmes perceptifs, Département d’études cognitives, 26909 École normale supérieure, PSL University, CNRS, Paris, France

**Keywords:** gloss, lightness, transparency, maximum likelihood conjoint measurement

## Abstract

The image intensity depends on the illumination, the reflectance properties of objects but also on the reflectance and absorption properties of any intervening media. In this study we present observers with glossy objects behind partially transmissive materials. The transparent layer causes an achromatic color shift and compression in luminance contrast, which can affect the perception of the specular reflections of the object behind the layer. In two distinct experiments, we examine how an achromatic color shift and the compression of luminance contrast affect perceived gloss. Thanks to the maximum likelihood conjoint measurement paradigm, we estimate the contamination of different transparent layers on perceived gloss. In the follow-up experiment, observers were asked to match the albedo and the gloss of surfaces seen in plain view to surfaces seen behind a transparent layer. Our results indicate a high degree of gloss constancy with some small but significant contribution of the transparent layer when estimating gloss, especially in the case of light-colored transparent layers. Overall, gloss is significantly overestimated.

## How to cite this article

Hansmann-Roth, S., & Mamassian, P. (2025). Perceiving gloss through transparency. *i–Perception*, *16*(4), 1–20. https://doi.org/10.1177/20416695251355381

## Introduction

The image intensity depends on the illumination, the reflectance properties of surfaces in the scene but also on the reflectance and absorption properties of any intervening media. Thus, all visual information is inherently ambiguous and estimating distal properties from the proximal stimulus is underconstrained. The visual system is constantly faced with the problem of reconstructing the three-dimensional scene from the two-dimensional retinal image to estimate for example material properties. Gloss is one optical property of many materials. Physical gloss describes the ability of a material to reflect light in a specular direction. Light that is specularly reflected from a surface leads to bright highlights on the surface that are known to evoke the perception of a shiny, glossy material. The brightest part in the image corresponds to the specular highlights, which are crucial to perceive a glossy surface (Beck & Prazdny, 1981). The intensity of specularly reflected light, however, is confounded with other variables such as the illumination, the surface geometry, the viewpoint of the observer and potential intervening media between the object and the observer. More recent studies showed that cues that are directly computable from the image serve as cues to judge gloss such as the geometry of highlights (Marlow & Anderson, 2013; Marlow et al., 2012; Qi et al., 2014) and also the contrast between the highlight and the surrounding non-highlight area serves as a proxy to judge the gloss of a surface. The importance of contrast gloss was already described earlier by Hunter (1937) as one of the six dimensions of gloss. Increasing the contrast between the highlight and the non-highlight area also increases perceived gloss. Therefore, a darker object appears glossier than a white or gray object, although the amount of specularly reflected light remains constant (Ferwerda et al., 2001; Hansmann-Roth & Mamassian, 2017; Hunter & Harold, 1987; Pellacini et al., 2000). For those objects, the albedo of the surface changes and subsequently the perception of gloss changes. Results from two other studies showed that dark backgrounds also increase perceived gloss (Doerschner et al., 2010; Hansmann-Roth & Mamassian, 2017). Moreover, perceived gloss also varies when an additional glossier material in placed on the same surface (Hansmann-Roth et al., 2017).

Decreasing the overall albedo of a surface affects perceived gloss through an increase in contrast, but the contrast between the highlights and the non-highlight area is also directly affected when placing an intermediate layer between the observer and the object. Depending on the transmission and the color of the transparent layer it induces an (1) an achromatic color shift and (2) compresses the overall luminance contrast of the object. However, judging gloss of a surface behind such a transparent layer should be invariant to such luminance changes *if* the transparent layer is fully accounted for.

**Figure 1.**
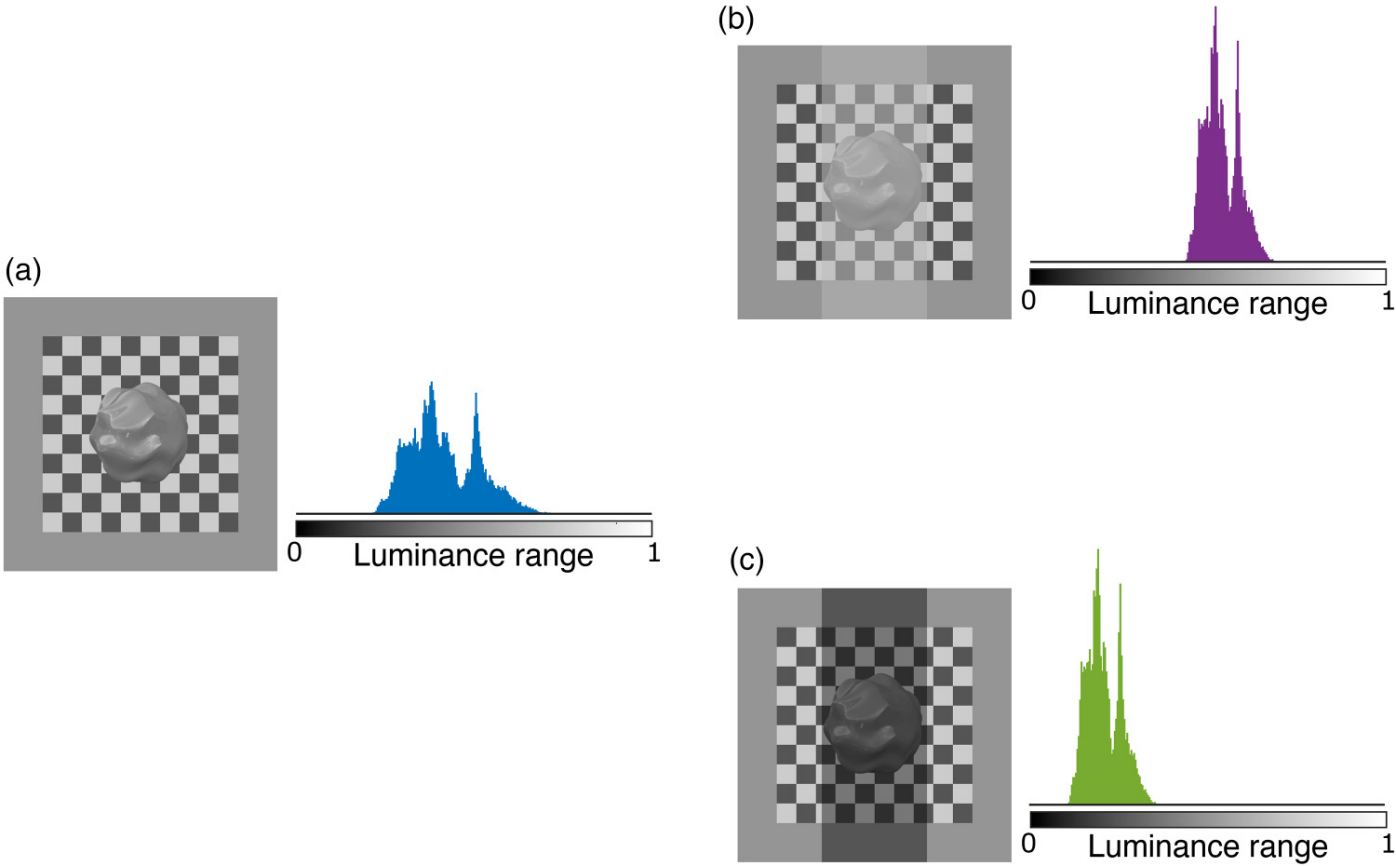
Example of the contributions of a transparent layer on the luminance histogram of an object. (a) Luminance histogram of an object seen in plain view. (b) and (c) Luminance histograms of objects seen through either a dark colored or a lighter colored transparent layer.

A very influential model of transparency was proposed by Metelli (1974a, 1974b) who developed his model by using an episcotister, namely a rotating disk with a missing segment in front of a bicolored background. Following this model, we illustrate in Figure 1(a) luminance histogram of an object in plain view and two luminance histograms of objects covered by either a dark or a light-colored transparent filter. The transparent filter shifts and compresses the luminance histogram, affecting the luminance of the brightest spot and the luminance contrast (luminance range). A light-colored transparent layer causes the histogram to shift towards higher luminance values (Figure 1(b)) and a dark transparent layer causes the luminance histogram to shift towards darker luminance values (Figure 1(c)). The role of the histogram shape was previously examined by Motoyoshi et al. (2007). They compared luminance histograms of matte and glossy images and showed that histogram skewness correlated well with apparent gloss. Images with positively skewed histograms appeared glossy. They found that skewness correlated well with perceived gloss, but later research shows that this holds only if the illumination geometry (light field) is held constant (e.g., Olkkonen & Brainard, 2010) and often fails when geometrical information is lost (Anderson & Kim, 2009; Kim & Anderson, 2010; Landy, 2007).

Studies on the perception of lightness (albedo of a surface) through transparency have shown that lightness perception of patches seen through transparency follow Metelli's model of transparency (Singh, 2004). This author used displays in which the transparent layer increased, decreased or preserved the mean luminance of the image. Results showed that perceived lightness through transparency depends on the luminance range and not the mean luminance in the region of transparency. Overall, a high degree of lightness constancy was obtained (between 78% and 96%) indicating that the visual system can compute material properties of the two overlying surfaces. Perceived transmittance of the transmissive filter, however, also depends on the reflectance of the transparent filter, contradictory to what has been initially proposed by Metelli (Robilotto et al., 2002; Singh & Anderson, 2002). Singh (2004) also showed that perceived transmittance varies with the mean luminance in the image. Three transparent layers with different reflectance were used, resulting in a different Michelson contrast for the different patches behind transparency: The patch behind a dark transparent layer yielded the highest Michelson contrast, whereas the patch behind the light transparent layer resulted in having the lowest Michelson contrast. Participants adjusted the luminance range of a texture behind the transparent layer to match a comparison display in plain view. Results showed that by changing the mean luminance of the patch behind transparency, perceived transmittance also changed. While Metelli's model of transparency predicts no variation of perceived transmittance as a function of mean luminance, perceived transmittance did vary with mean luminance. These matching results are consistent with the relative contrast within the region of transparency and therefore, Singh (2004) proposed a Michelson contrast ratio model that better explained their matching results. Both lightness matching and transmittance matching of the transparent filter can exhibit deviations from Metelli's model of transparency (Singh & Anderson, 2002, 2006).

**Figure 2.**
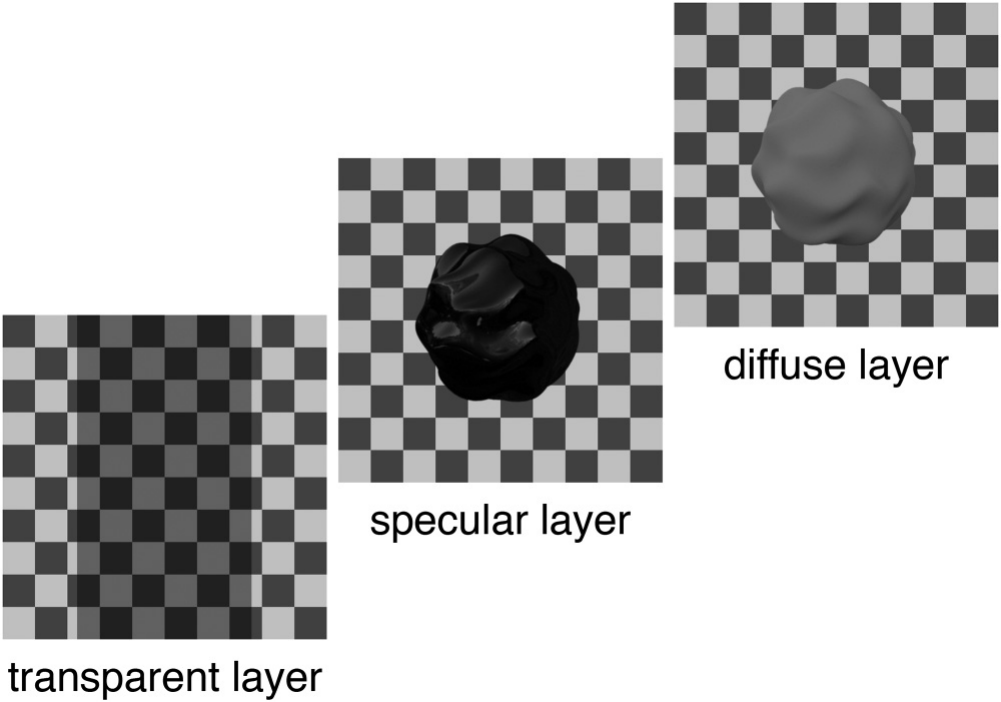
Sketch of the different image layers of a glossy object behind transparency.

All the aforementioned experiments used simple flat surface patches as targets. Therefore, matching corresponded to a single reflectance value only. Most natural objects, however, exhibit a much more complex luminance histogram due to shading and shadows (Kawabe, 2019) as well as additional specular reflections (see Figures 1(a) and 2). Figure 1(b) and (c) illustrates the changes in the luminance contrast when an object is placed behind a transparent layer. Figure 2 illustrates again the different layers that a complex glossy object behind transparency is composed of. To estimate the albedo of the object, observers have to account for the changes in the image introduced by the specular layer and the transparent layer. To estimate the gloss of the object, observers have to account for the diffuse and the transparent layer.

In the current study, we attempt to study these complex interactions between gloss, albedo and transparency. In particular, we investigate the role of luminance contrast in gloss perception and measure observers’ degree of lightness and gloss constancy through different transparent layers. Instead of manipulating luminance contrast through manipulations of the surface albedo, we introduced transparent layers to induce changes to the luminance contrast.

We used complex bumpy objects that have a particular shading pattern and are made from glossy materials. In Experiment 1, we use a paired-comparison task to investigate whether the transparent layer is contributing to observers’ gloss judgment. We then use the method of maximum likelihood conjoint measurement (MLCM) to quantify any influence induced by the transparent layer. MLCM has successfully been used to study the mutual interactions of various visual features on perception (Chadwick et al., 2018; Gerardin et al., 2018; Hansmann-Roth & Mamassian, 2017; Ho et al., 2008; Rogers et al., 2016). In Experiment 2, observers could control the albedo and gloss of objects to match the material of the same objects seen behind transparency to objects seen in plain view.

## Experiment 1

### Materials and Methods

#### Stimuli

We rendered four different stimuli with varying levels of specularity and a constant gray albedo. A sphere was perturbed using sine wave modulations to crease a bumpy shape (see Dövencioğlu et al., 2017). Stimuli were rendered with Mitsuba 0.5.0 using the RenderToolbox3 package for MATLAB (Heasly et al., 2014; see also http://rendertoolbox.org). RenderToolbox acts as an interface to the Mitsuba rendering software (Wenzel, 2010; see also www.mitsuba-renderer.org). Stimuli were rendered using the Ward model (Ward, 1992). The Ward model defines surface reflectance by three different parameters: ρ_d_ controls the diffuse reflection or albedo, ρ_s_ controls the strength of the specular reflection, and α controls the spread of the specular lobe. The values ρ_d_ and α were kept constant at 0.1 and 0.005, respectively. We manipulated the strength of the specular component to simulate different levels of specularity (*ρ*_s_ = 0.02, 0.035, 0.06, and 0.13). Values were chosen by eye so that differences between levels were approximately perceptually equal. All stimuli were illuminated with an environment map (Hallstatt, downloaded from http://dativ.at/lightprobes/). We cut out the rendered objects from their original background and placed them in front of a checkerboard background consisting of dark and light gray checks. Images were converted from RGB to gray scale in MATLAB. MATLAB uses the following conversion weights: 0.2989 × R + 0.5870 × G + 0.1140 × B. The transparent layers were also generated in MATLAB using Metelli's episcotister model by converting the luminance pixel using the following equation

(1) p=αb+t(1−α)

where *p* is the luminance value in the region of transparency. The parameter *t* corresponds to the reflectance of transparent filter when it is opaque, *α* refers to the transmittance of the transparent layer and *b* refers to the luminance of the background pixel. We presented each stimulus behind four different transparent layers that varied in their reflectance *t* while *α* was held constant at 0.5. The reflectance of the four different layers ranged from black to white with two intermediate gray levels in between (*α* = 0, 0.33, 0.66 and 1). A single stimulus including its checkerboard subtended 12.1 × 12.1 degrees of visual angle on the LCD screen while the single object subtended 6.2 × 6.2 degrees of visual angle.

#### Apparatus

All stimuli were displayed on a 24-inch calibrated LCD monitor (ViewSonic V3D245, ViewSonic Corporation, Brea, CA) with a linearized gamma. The resolution was set to 1920 × 1080. All stimuli were displayed using MATLAB R 2010a and Psychtoolbox-3 (Brainard, 1997) that ran on a MAC Pro Quadro-Core Intel Xeon with OSX 10.5.8.

#### Procedure

All participants were seated with their head placed in a chin rest positioned 57 cm in front of the screen and observed the stimulus with their dominant eye while the other eye was occluded using an eye patch. The eye dominance was assessed for each observer using the Miles test (Miles, 1930). The experiment took place in a dark room. Two stimuli were presented simultaneously on the left and right side of the fixation cross. The four gloss levels and the four different transparent layers resulted in 120 possible combinations without self-comparisons. Participants ran 480 trials in total (four repetitions per combination). Each trial started with a fixation cross for 300 ms followed by the presentation of the two comparison stimuli. On each trial, participants were prompted to indicate which surface appeared to be made from a glossier material. After pressing the appropriate response key, the two stimuli disappeared, and the next trial began.

#### Observers

Eight observers (*M*_age_ = 28.9, three females) participated in the first experiment. All participants were naïve to the purpose of the study and had all normal or corrected-to-normal vision. They all gave written, informed consent. All experiments were done in agreement with the local ethics committee from École Normale Supérieure and the Declaration of Helsinki.

#### Analysis

The goal of this experiment was to evaluate whether the reflectance of the transparent layer influences observer's gloss judgments. To describe and quantify any contribution of this unattended feature we used MLCM.

We analyzed our data using MLCM package (Knoblauch & Maloney, 2012, 2014) in the open-source software R (R Development Core Team, 2012) to estimate the perceptual scale values and model the contribution of specularity and the reflectance of the transparent layer on perceived gloss. The methods used in this study were the same as those of Ho et al. (2008) and Hansmann-Roth and Mamassian (2017) and are reproduced here for the reader's convenience.

We used the framework from Ho et al. (2008) considering three different models. The independent observer model predicts that only the physical dimension of interest (specularity) can influence the perception of that dimension (perceived gloss). The second model, the additive observer model predicts that the irrelevant dimension (reflectance of transparent layer) can influence perceived gloss. The strength of this influence, however, is independent of the specularity of the object. The full/saturated observer model allows for an additional interaction term between the dimension of interest and the irrelevant one.

Each of the two objects presented during one trial have a fixed specularity level $\varphi_{i}^{g}$ , $\varphi_{k}^{g}$ and the transparent layer has a fixed reflectance level $\varphi_{j}^{r}$ , $\varphi_{l}^{r}$ . When observers indicate which object appears glossier, an estimate of perceived gloss $\psi^{g}$ is computed for both surfaces. This perceptual estimate depends on the perceived gloss and potentially on the perceived reflectance of the transparent layer

(2) $$\;\psi_{\mathrm{ij}}^{g}=\psi_{i}^{g}+\psi_{j}^{r}$$

Both estimates are then compared, and the difference is computed

(3) $$\Delta (i,j,k,l)=(\psi_{i}^{g}+\psi_{j}^{r})-(\psi_{k}^{g}+\psi_{l}^{r})+\varepsilon$$

where ε is an unbiased normally distributed judgment error: $\varepsilon \;\;∼\;\;N(0,\;\sigma^{2}$ ).

For each material, the difference is computed separately and then the differences are added together to make a final decision. For each reflectance and specularity level, we need to estimate the parameters $\psi_{i}^{g}$ and $\psi_{j}^{r}$ from the data. If the reflectance of the transparent layer has no additional influence on perceived gloss, the independent observer model best describes the data

(4) $$\Delta (i,j,k,l)=\psi_{i}^{g}-\psi_{k}^{g}+\varepsilon$$

For a full model, perceived glossiness is modeled with an interaction factor

(5) $$\Delta (i,j,k,l)=(\psi_{i}^{g}+\psi_{j}^{r}+\psi_{\mathrm{ij}}^{\mathrm{gr}})-(\psi_{k}^{g}+\psi_{l}^{r}+\psi_{\mathrm{kl}}^{\mathrm{gr}})+\varepsilon$$

All three models are nested within each other and were tested against each other using a likelihood ratio test of nested models following the $\chi^{2}$ -statistic. Further details for this procedure can be found in Ho et al. (2008), Knoblauch and Maloney (2012, 2014) and Hansmann-Roth and Mamassian (2017).

### Results

In the first experiment, observers were presented with two objects behind a transparent layer and they were asked to indicate which object is made out of a glossier material. Figure 1 plots the raw data in a matrix format. Each cell corresponds to a combination of stimulus pairs and the intensity corresponds to the proportion of times that the object O*_kl_* is perceived as having a glossier material than object O*_ij_*.

**Figure 3.**
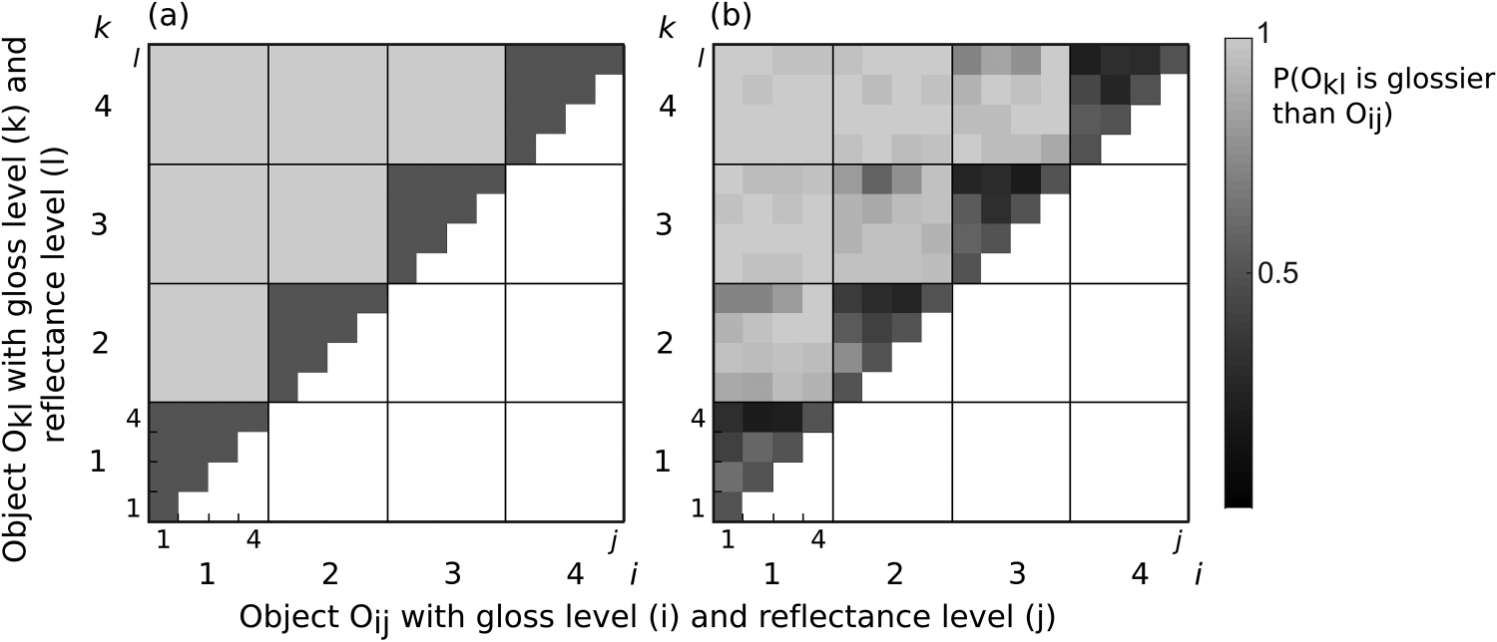
Conjoint proportion plots: Each diagram plots the proportion of the object O*_kl_* being perceived as made from a glossier material than O*_ij_*. The gloss levels *k* and *i* are indicated by the large squares (large numerical labels) and each large square is subdivided into smaller 4 × 4 squares, indicating the reflectance levels *j* and *l* of the transparent layer (small numerical labels). (a) Prediction of an observer that is unaffected by the reflectance of transparent layer (the secondary dimension *j* or *l*). An ideal observer always chooses the glossier object O*_kl_* over O*_ij_*. (b) Results averaged across all eight observers. The gradient found within the smaller squares shows that if object O*_ij_* is presented behind a darker transparent layer, it is chosen more often to be made from a glossier material than object O*_kl_* although their specularity is the same.

Figure 3(a) plots a simulation of an independent observer model. The independent observer model predicts that the reflectance of the transparent layer has no influence on the gloss judgment. Figure 3(b) plots the raw data averaged across all eight participants. We found deviations from a gloss constant observer. The influences of the different transparent layers are strongest when the levels of specularity of the two objects were similar (large squares above the diagonal). Lighter colored layers reduced perceived gloss of the objects (upper half of the big squares are darker than the lower half).

**Figure 4.**
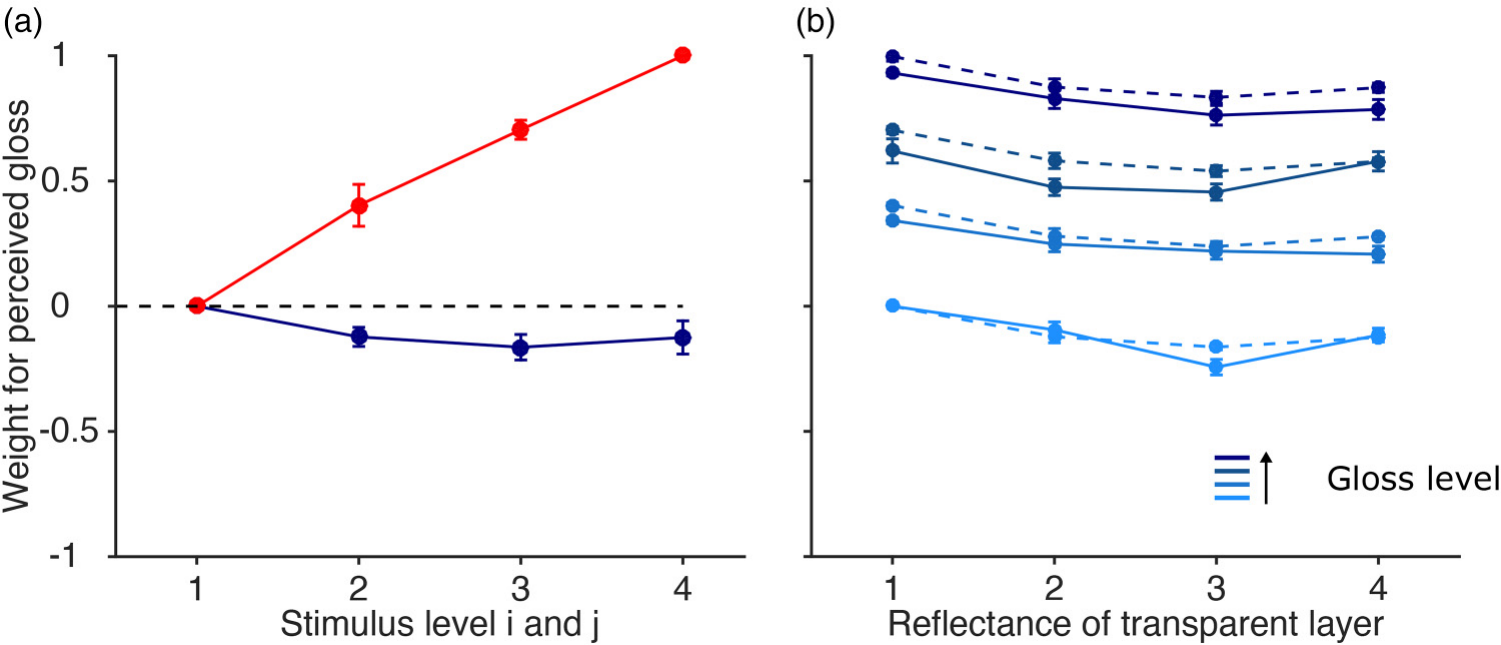
(a) Normalized parameter estimates obtained from fitting the data with the additive model. Parameter estimates for perceived gloss are plotted against the stimulus level for either the specularity or the reflectance of the transparent layer. The red curve corresponds to the contribution of specularity and the blue curve corresponds to the contribution of the different transparent layers. (b) Normalized parameter estimates obtained from fitting the data to the additive model (dashed line) and the full model (solid lines). Parameter estimates are averaged across all participants. Error bars correspond to ±1 SEM.

Figure 4(a) plots the parameter estimates obtained from fitting the additive model to the data. Parameter estimates for each level of specularity and each transparent layer are averaged across all participants. The parameter estimates were normalized to the maximum value for each observer to better describe the relative contribution of the unattended feature. The red line corresponds to the estimates of the primary dimension. Obviously, specularity correlates well with perceived gloss. The blue line corresponds to the parameter estimates of the secondary dimension. We analyzed whether the secondary dimension contaminates our gloss judgments. Therefore, the fits of the independent model are compared to the fits of the additive model. In the independent model the parameters of the secondary dimension are set to zero. A nested hypothesis test revealed that, at the Bonferroni-corrected level (*p* = .0063), we had to reject the independent model for six out of eight participants (*p* < .002). This shows that the secondary dimension can indeed contribute to observer's gloss judgments. Perceived gloss was reduced by about 16% when objects were presented behind lighter colored transparent layers. The additive model predicts that the influence of the transparent layer is independent of the specularity of the object. To test whether our data follows this prediction we fitted the full model to our data and statistically compared it with the fits obtained from the additive model. Figure 4(b) visualizes the comparison between the additive and the full model. The dashed lines correspond to the fit from the additive model and the solid lines contribute to the fits from the full model. A nested hypothesis test comparing the models revealed that at the Bonferroni-corrected level (*p* = .0063), we had to reject the full model for all our eight participants. Therefore, we can retain the simpler additive model. The influence of the transparency was independent of the specularity.

### Discussion

The presented study investigated observer's ability to perceive gloss and albedo of complex objects through transparency. The results obtained from the experiment verified that different transparent layers could affect observer's gloss judgments. In particular, we examined how the contribution of the transparent layers differed for different reflectances. The different reflectances of the layer shift the luminance histogram to higher or lower ranges. In the first study we used a paired-comparison task and analyzed the data using maximum likelihood conjoint measurements. We quantified the strength of the contamination of the transparent layer on perceived gloss. Our data revealed that perceived gloss was reduced by about 16% when the glossy object was presented behind lighter colored transparent layers. Since we did not include an object in plain view, we are not able to precisely quantify the degree of gloss constancy. Therefore, in the next experiment we reexamined observers’ ability to estimate gloss behind transparency using a matching task. Participants were asked to directly match material properties of objects seen in plain view to objects seen behind transparency.

## Experiment 2

### Materials and Methods

#### Stimuli

**Figure 5.**
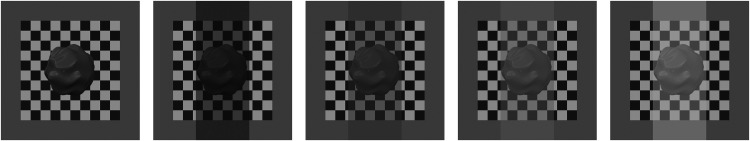
Overview of the four different transparent layers used in the experiment. The object (Shape 1) on the left in presented in plain view and the other four objects are identical but presented behind different transparent layers.

We tested eight different stimuli as targets (two different specularity levels: *ρ*_s_ = 0.01 and *ρ*_s_ = 0.028, two different albedos: *ρ*_d_ = 0.1 and *ρ*_d_ = 0.2 and two different shapes). They were presented behind four transparent layers (Figure 5) that had different reflectance values (constant *t* = 0.5, *α* = 0.2, 0.4, 0.6 and 0.8).

**Figure 6.**
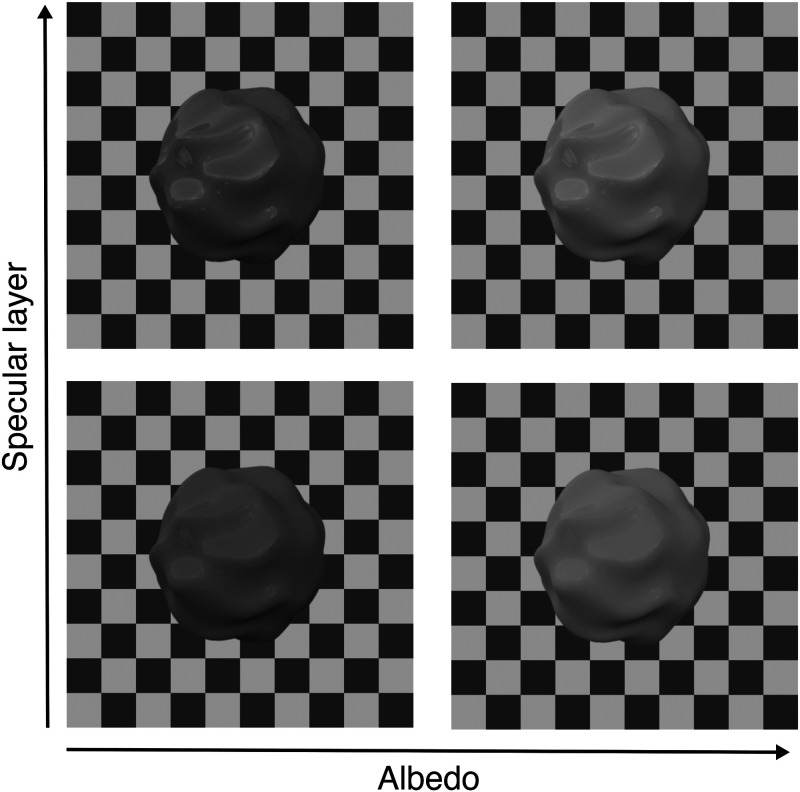
Examples of the four different materials (2 albedos × 2 gloss levels) for shape #2.

Figure 6 shows all four different materials used in the experiment. The two objects within a row differ in their albedo and the two objects in a column differ in their amount of gloss.

**Figure 7.**
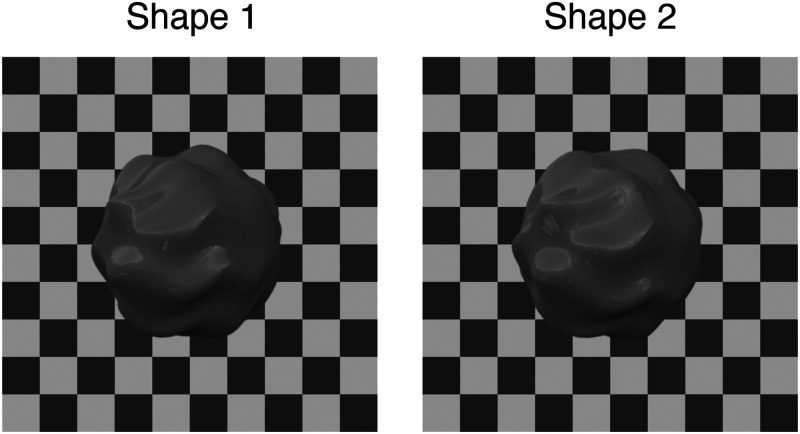
Examples of the two different shapes.

We used two different shapes. One shape was identical to the shape in Experiment 1 and the second shape was simply a rotated (10-degree rotation along the vertical and 13 degrees along the horizontal axis) version of the first shape (Figure 7).

For the matching task, we pre-rendered all 49 stimuli that participants could select from. Stimuli varied in gloss and albedo on a seven-step scale. All albedo and gloss levels were chosen such that two consecutive levels were approximately perceptually equal. Since perceived gloss is known to follow a non-linear relationship, we used a maximum likelihood difference scaling (MLDS; Maloney & Yang, 2003) dataset to scale the gloss levels. The seven different levels of albedo were also selected so that the differences were approximately equal. MLDS allows us to estimate the relationship between physical and perceptual dimensions. Based on the fitted physical-to-perceptual transformation, the appropriate rendering values were selected, and all 49 objects were rendered. Targets were selected directly from this set. All stimuli were rendered using the RenderToolbox3 in MATLAB R 2014b as an interface to Mitsuba (Version 0.5.0). Targets and matching stimuli were cut out from their background and placed in front of checkerboard backgrounds. The different targets were covered with the different transparent layers, while the matching stimuli were presented in plain view in front of the checkerboard. To ensure a constant contrast between the object and the checkerboard background we changed the albedo of the checks of the matching stimulus each trial to match it to the luminance of the checks behind the transparent layer. The shapes of the target and the matching stimulus were always dissimilar. If the target was shape 1, then the matching stimulus was automatically shape 2 and vice versa. This prevented observers from simply matching the luminance between stimuli.

#### Apparatus

The setup was identical to the setup in the first experiment.

#### Procedure

All participants were seated with their head placed in a chin rest positioned 57 cm in front of the screen and observed the stimulus with their dominant eye while the other eye was occluded with an eye patch. Eye dominance was again assessed using the Miles test (Miles, 1930). The experiment took place in a dark room. Participants performed a matching task in which they were asked to match the material properties of an object in plain view so that it matched the material properties of an object seen behind transparency.

Participants started with a familiarization phase consisting of 30 trials to acquaint themselves with the matching procedure. Each participant passed three different conditions. In one condition participants were asked to match the albedo of the object (Condition 1) seen in plain view to the object seen behind transparency while physical gloss of the object was held constant at the correct intensity. In a second condition, participants were asked to match the gloss of the object (Condition 2), while the albedo was held constant and in a third condition participants were asked to match both material properties simultaneously (Condition 3). Each condition was tested in separate blocks. Before each block, participants were informed about the condition. Participants ran six blocks in two separate sessions on two different days. Each session lasted about one hour. We randomized conditions so that the first condition was either a condition in which gloss or albedo was fixed.

During each trial the target (behind the transparent layer) was presented on one side of the screen and a random matching stimulus on the other side of the screen. Participants could change the albedo and/or the gloss (depending on the condition) until they were satisfied with their match. More specifically, they could increase or decrease the albedo and the specular intensities. One pair of buttons decreased/increased the diffuse component, and another pair of buttons decreased/increased the specular component. If they exceeded the range of matching possibilities, they were informed by an auditory beep. Once the observer was satisfied with the match, they confirmed their selection by pressing the space bar and the next trial began. All blocks were randomized.

#### Observers

A total of eight observers (*M*_age_ = 29.9, six females, five observers from Experiment 1) participated in this experiment. All observers were naïve to the purpose of the study and had all normal or corrected-to-normal vision. They all gave written, informed consent. All experiments were done in agreement with the local ethics committee from École Normale Supérieure and the Declaration of Helsinki.

### Results

In the second experiment, participants were asked to match the material of an object seen in plain view to the material of an object seen behind transparency.

#### Analysis

For the forthcoming analysis, we calculated a matching error based on the seven-step matching space, in which the steps correspond to equal perceptual differences. We plot the matching error as the difference between the correct material value and the matched material value. Negative values correspond to an underestimation of albedo and/or gloss and positive values correspond to an overestimation of albedo and/or gloss. In Conditions 1 and 2, we kept one of the material properties constant. Moreover, we varied the shape between the target and the matching object. In an initial step of the analysis, we examined whether the second material property or the shape influenced the matching behavior using paired *t*-tests. To further assess the influence of the transparent layer and participant's degree of constancy we conducted three-way repeated-measures ANOVA, with Greenhouse–Geisser corrections, where applicable, after testing for sphericity using Mauchly tests. ANOVAs were conducted in the open-source software R (R Development Core Team, 2012) using a random effects model from the *ez* package (Lawrence, 2016).

#### Albedo Matching (Condition 1)

In Condition 1, participants were asked to match the albedo of the object while the specularity was already set to its correct value. Moreover, we used two different shapes and two different specularities. To examine whether these manipulations influenced observers’ matching ability we compared the mean matching error between the two different shapes and specularities. For the two different specularities, a paired *t*-test revealed that the matching error was indeed affected by the specularity of the object: *t*(7) = 6.42, *p* < .001 and a second paired *t*-test revealed that albedo matching was independent of the shape of the object: *t*(7) = −0.15, *p* > .05.

**Figure 8.**
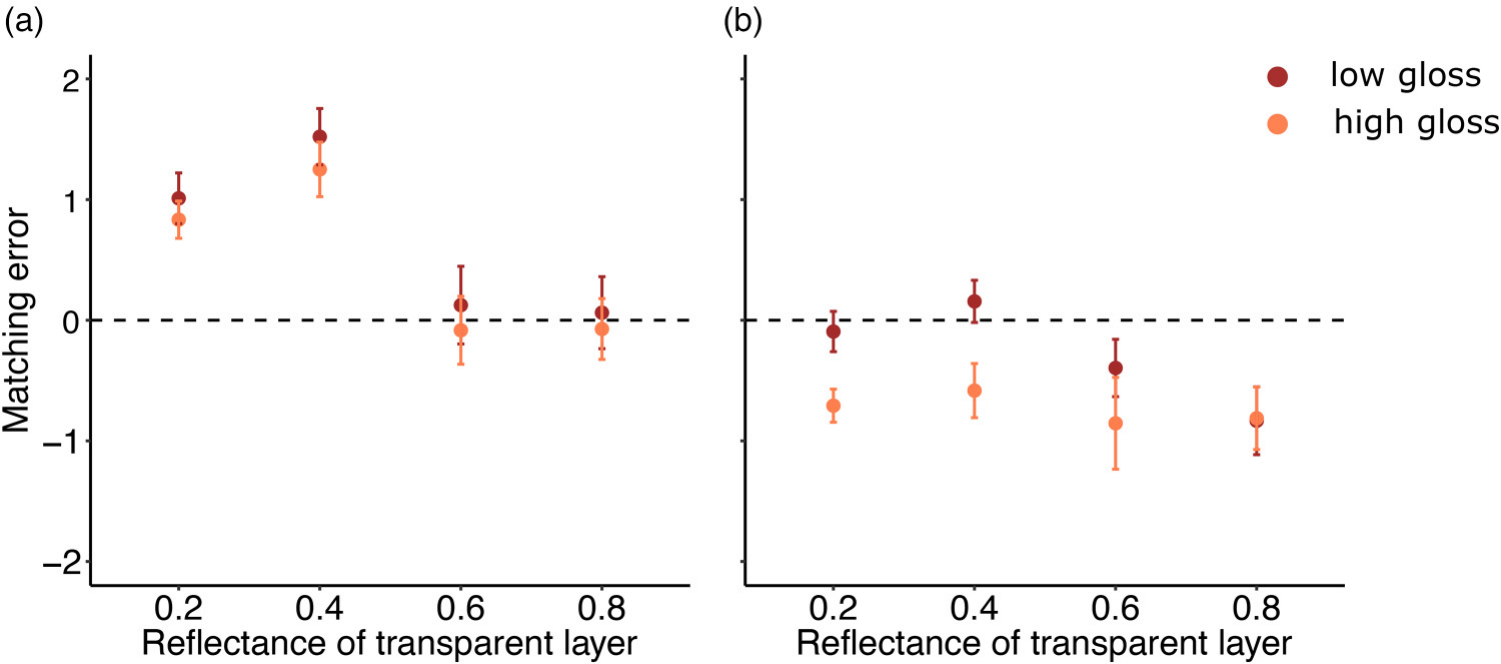
Matching error in condition 1 plotted against the four different transparent layers. Each color corresponds to one of the two different gloss levels used as target materials. (a) Plots the matching error against the four different transparent layers for a dark-colored object. (b) Plots the matching error for a lighter-colored object. Error bars correspond to ±1 SEM.

In a second step, we analyzed whether the transparent layers introduced into the scene affected perceived albedo and whether these effects depend on the reflectance of the transparent layer. As visible from Figure 8 the overall matching errors for the two albedos were higher for the two darker transparent layers compared to the two lighter transparent layers. The overall darkening or lightening of the surface was taken into account. However, sometimes observers overcompensated for this darkening/lightening as visible by the higher matching errors (towards lighter albedos) for the dark objects. Moreover, there was no large difference between the two different gloss levels (most orange and red datapoints are overlapping). A three-factor (4 transparent layers × 2 albedos × 2 specularities) repeated-measures ANOVA confirmed these observations and revealed a main effect of transparency, *F*(3, 21) = 10.24, *p* < .01, *η^2^* = 0.28, a main effect of albedo, *F*(1,7) = 77.45, *p* < .001, *η^2^* = 0.41, and a main effect of specularity: *F*(1, 7) = 41.21, *p* < .001, *η^2^* = 0.06. Moreover, we found significant interactions between the transparency and albedo [*F*(1.71, 11.94) = 20.61, *p* < .001, *η^2^* = 0.078] and between transparency and specularity [*F*(3, 21) = 6.27, *p* < .01, *η^2^* = 0.02]. We found no significant interaction between albedo and specularity [*F*(1, 7) = 5.87, *p* = .05] and the three-way interaction yielded no significant results. Matching the albedo of the object is influenced by the reflectance of the transparent layer, the specularity of the object and the matching error also differs for different albedos.

#### Specularity Matching (Condition 2)

When participants were asked to match the gloss of the object seen behind the transparent layer, we simultaneously manipulated the shape and the albedo of the object. To investigate any influence of the albedo and the shape on gloss matching we compared the mean matching error between the two shapes and the two albedos, respectively. For the two different albedos, a paired *t*-test revealed that the matching error was not affected by the albedo of the object: *t*(7) = 2.27, *p* > .05. The shapes of the target and the matching object were always dissimilar. If the target was an object with shape 1, the matching object was automatically set to shape 2 (Figure 7). Therefore, we tested whether the shape affects observers’ matching behavior. A paired *t*-test revealed that the shape affected indeed the matching error: *t*(7) = −5.68, *p* < .001.

**Figure 9.**
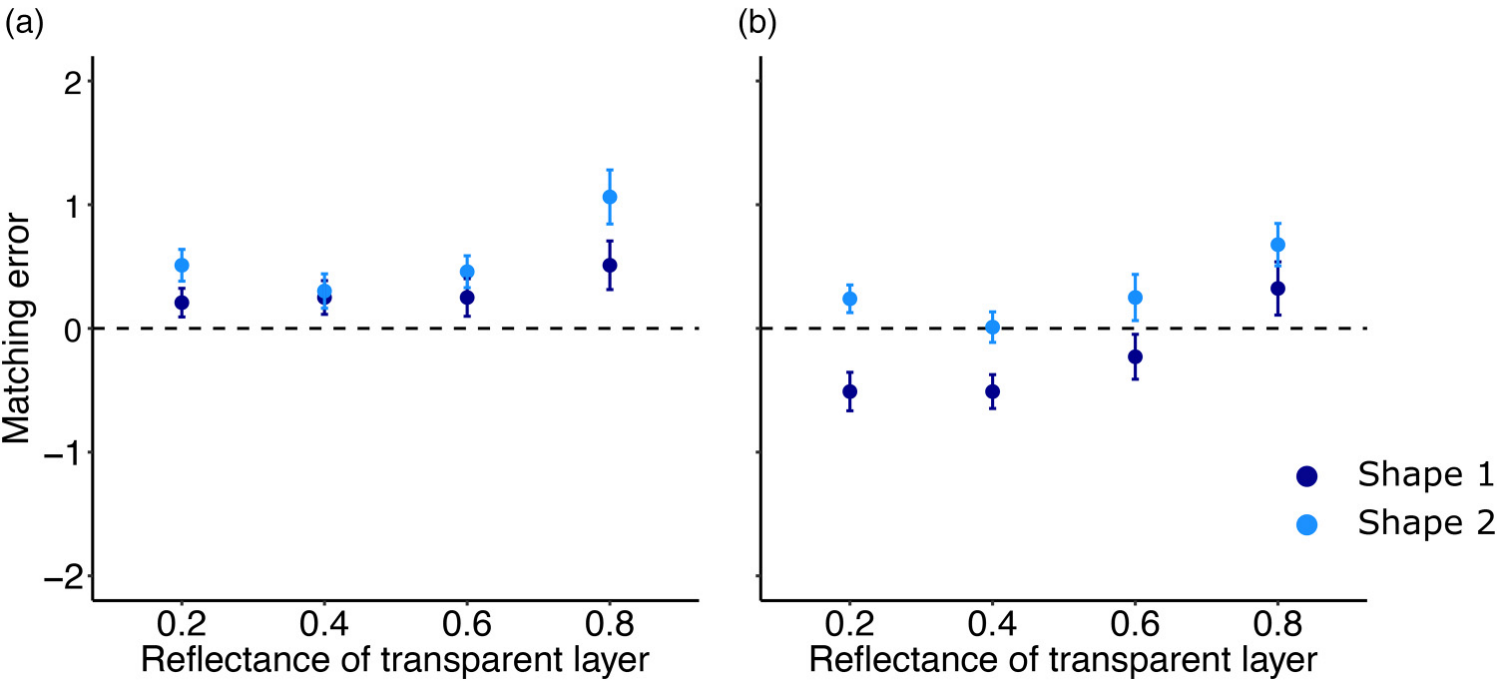
Matching error in Condition 2 plotted against the four different transparent layers. Each color corresponds to one of the two different shapes used as targets. (a) Matching error against the four different transparent layers for a low gloss object as seen in Figure 6 (bottom-left object). (b) Matching error for a highly glossy object as seen in Figure 6 (top-left object). Error Bars Correspond to ±1 SEM.

Our main question was to investigate whether the transparent layer in front of the object and in particular, whether the reflectance of the transparent layer influences participants’ matching ability. Overall matching errors were quite small, and we found the same pattern for the low and high gloss object (compare Figure 9(a) and (b)). The object shape influenced the accuracy of observers’ gloss matching behavior. It appears that shape 2 was perceived as glossier than shape 1 (light blue data points above the dark blue data points). A three-factor (4 transparent layers × 2 shapes × 2 specularities) repeated-measures ANOVA confirmed these observations and revealed a main effect of the transparent layers, *F*(3, 21) = 33.83, *p* < .001, *η^2^* = 0.25, a main effect of shape, *F*(1, 7) = 32.28, *p* < .001, *η^2^* = 0.18, and a main effect of specularity: *F*(1, 7) = 7.013, *p* < .05, *η^2^* = 0.19. None of the two-way interactions were significant. However, the three-way interaction yielded in a significant result: *F*(3, 21) = 6.221, *p* < .01, *η^2^* = 0.025. The nature of this small but significant interaction is shown in Figure 9: The effect of shape on the matching behavior was the strongest for dark transparent layers when the object is made from a glossy material (Figure 9(b)). Shape 2 was on average perceived as being made from a glossier material.

**Figure 10.**
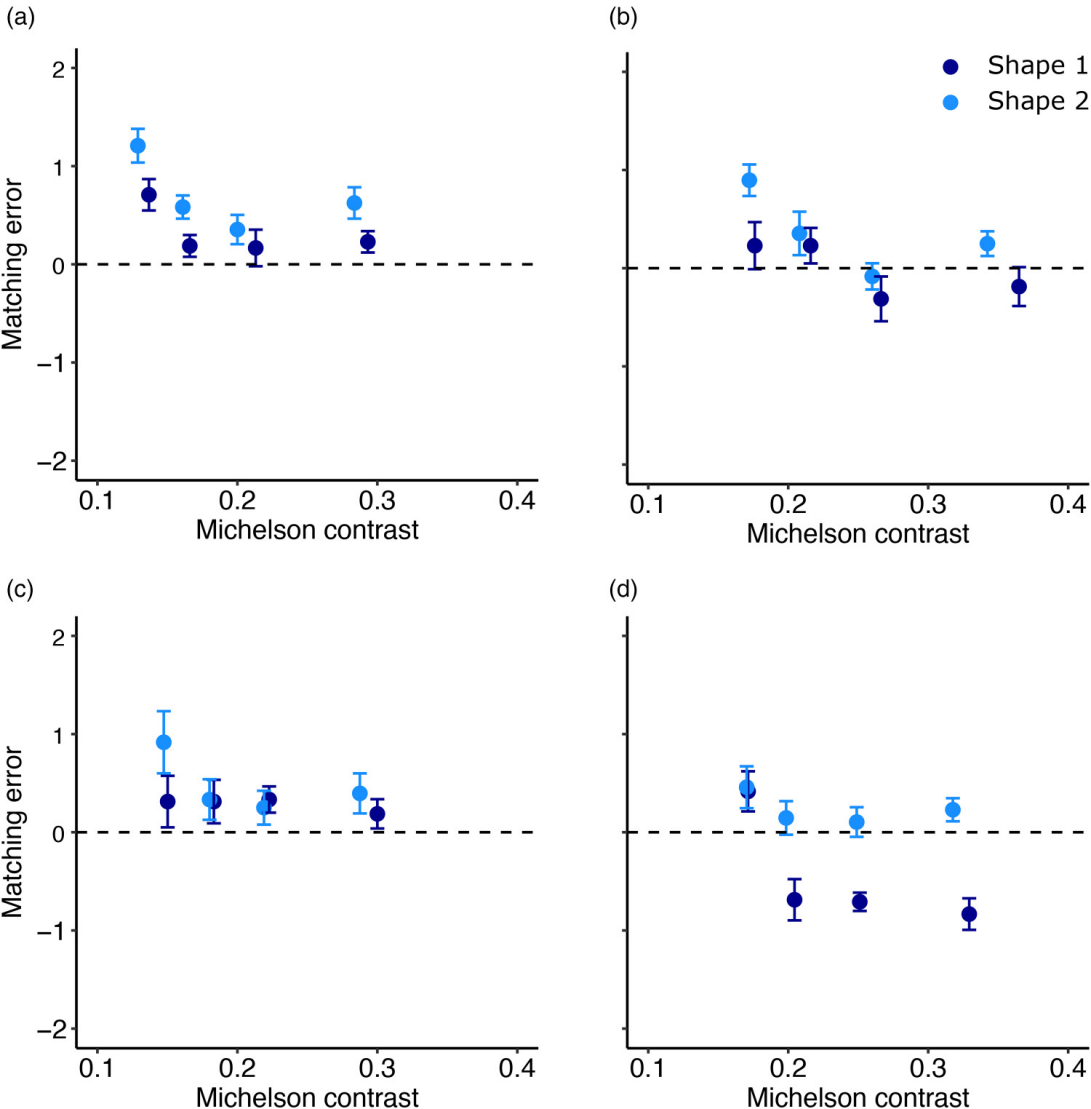
Matching error in Condition 2 plotted against the Michelson contrast of the object behind the four transparent layers. Each color corresponds to one of the two different shapes used as targets. (a) Matching error for low gloss and dark object. (b) Matching error for a highly glossy and dark object. (c) and (d) Matching error for lighter colored object with low gloss (c) or the highly glossy (d) object. Error bars correspond to ±1 SEM. (While the albedo of the object did not contribute to the matching errors, we separated it here, since the Michelson contrast is not identical for objects that have the same specularity level and shape but differ in albedo).

In Figure 10, we plot the matching error of the second condition again but as a function of Michelson contrast. Contrast is known to be an important feature to estimate gloss. Observers could have also attempted to match the contrast of the test image and the comparison image. Since the light and dark colored object have a different contrast, we separated the matching error in Figure 10 by albedo as well. The results from Figure 10 exhibit a similar pattern as in Figure 9. The image with the lowest contrast (corresponding to the image covered by the lightest transparent layer) results in the most positive matching error, while the matching error gradually decreases with an increase in contrast. The objects with the lowest contrast show the largest matching error. Observers tended to compensate for the larger reduction in contrast by increasing the specular reflectance of the matching stimulus resulting in a positive matching error.

#### Matching Both Material Properties (Condition 3)

**Figure 11.**
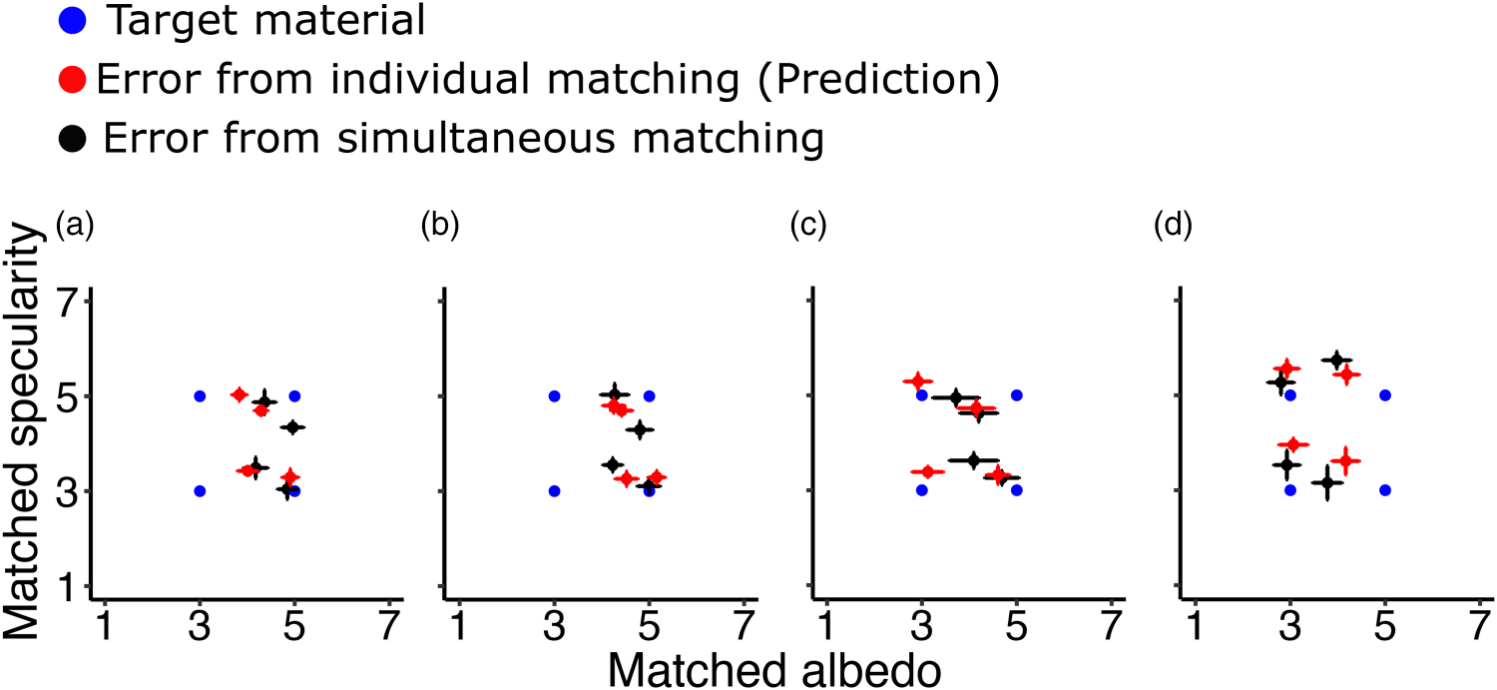
Matching results of Condition 3 plotted into the 7 × 7 matching space (black dots). Matching errors from Conditions 1 and 2 are plotted against each other (red dots) and additionally the materials of the four targets are plotted in blue. Each diagram corresponds to one of the four transparent layers. Matching results are averaged across all participants. Error bars correspond to ±1 SEM. (a) Matching results when the target was presented behind the darkest transparent layer. (b) Matching results for the second darkest transparent layer. (c) and (d) Matching results for the lighter-colored transparent layers.

In the final third condition, participants were asked to match both material properties simultaneously. Based on the data we obtained from the first two conditions we can predict the amount of deviation from constancy assuming that the size of the matching errors is identical to the first two conditions. Figure 11 plots the four different targets (two gloss levels, two albedo levels) into the 7 × 7 matching space (blue dots). Each diagram corresponds to one of the transparent layers. Red dots correspond to the matching error from the first two conditions and black dots represent the error obtained from the third condition. Overall, matching errors when both, albedo and specularity had to be reproduced resembled the individual matching errors from Conditions 1 and 2 (red and black dots are at similar values in Figure 10). Observers could simultaneously reproduce the gloss and albedo levels of the objects. To better evaluate whether the size of error was actually similar we compared the Euclidean distance from the *correct material* (blue dot) to the matched material (red dot) from the first two conditions to the Euclidean distance between the correct material and the matched material (black dot) from the third condition. The combined error from the first two conditions serves as a prediction for the error in the third condition.

**Figure 12.**
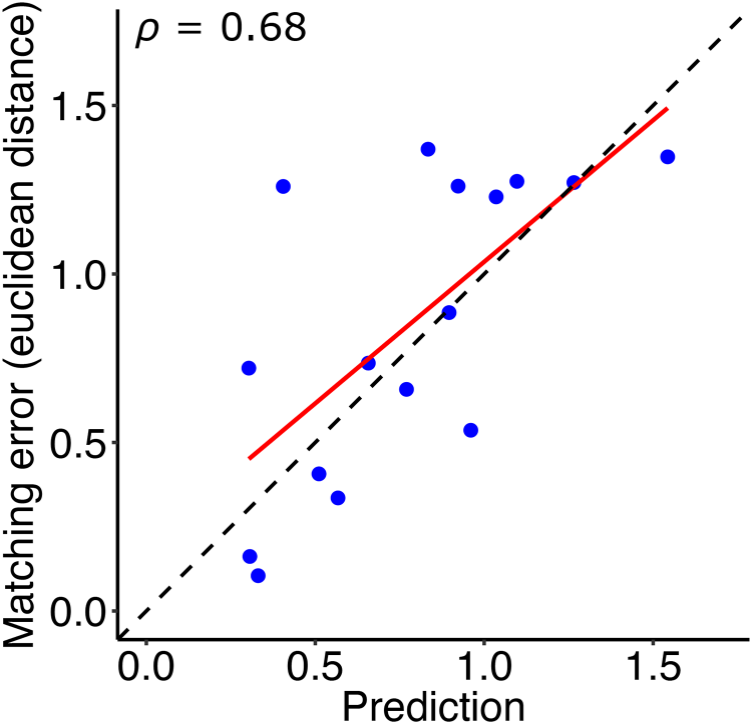
(a) Matching error as the Euclidean distance between the matched material and the actual material of the target plotted against the predicted Euclidean distance from the individual matching of albedo and gloss.

A simple linear regression was calculated to predict the matching error in Condition 3 based on the individual matching in Conditions 1 and 2. We found a significant correlation between the matching error in the third condition and the predicted error based on the first two conditions: *r*(14) = .68, *p* < .01 (Figure 12). The slope (*b* = 0.84) was statistically tested against 1.0 with a one-sample *t*-test and did not deviate significantly from 1 [*t*(15) = −0.662, *p* = .52].

### Discussion

In the second experiment, we investigated participants’ ability to estimate gloss behind transparency using a matching task. Observers were asked to directly match material properties to objects seen in plain view to objects seen behind transparency. In one of the conditions, observers matched the gloss of the object only, while the albedo of the matching object was fixed, in the other condition, observers matched the albedo of the object while the specularity was fixed and in the third condition both properties were matched simultaneously.

#### Specularity Matching

Our results from the specularity matching corroborated the results from the first experiment. Overall, participants’ matching ability was quite high. However, we found significant contributions of the transparent layer. The matching error was more positive when the object was behind the light-colored transparent layer. Consequently, the light-colored transparent layer must have reduced perceived gloss more than the darker colored layers resulting in an overshoot. Metelli already noted that lighter colored transparent layers appear more opaque than darker transparent layers. Singh and Anderson (2002) confirmed these observations. Their observers systematically underestimated the transmittance of the light-colored transparent layer and overestimated the transmittance of the dark colored layer.

Therefore, in the second step, we analyzed the matching error as a function of the contrast in the image (Michelson contrast of the object with the transparent layer in front). Objects behind the lighter colored transparent layer exhibit the lowest Michelson contrast and objects behind darker colored transparent layers have a higher contrast. The objects with the lowest contrast show the largest matching error. Observers tended to compensate for the larger reduction in contrast by increasing the specular reflectance of the matching stimulus resulting in a positive matching error. It appears that the simple cues such as Michelson contrast are used to estimate and match the glossiness of the object. Contrast strongly affects perceived gloss (Hansmann-Roth & Mamassian, 2017; Hunter & Harold, 1987; Pellacini et al., 2000). The same object behind different transparent layers varies in contrast and observers’ matching error are larger when this contrast reduction is stronger.

Interestingly, this matching error also varied with the shape of the target and the matching stimulus. The shapes of the target and the matching stimulus within a single trial were always different. Even though we only rotated the object before rendering, one shape seems to exhibit larger and/or more pronounced highlights than the other shape (Figure 7). Hence, these objects were perceived as being made from a slightly glossier material. The position and shape of specular highlights depend largely on the underlying shape. Experiments have shown that changes in the shape of the objects can be perceived as changes in the material (Nishida & Shinya, 1998; Olkkonen & Brainard, 2011; Vangorp et al., 2007; Wendt et al., 2010). Highlight features like their brightness, contrast, or the amount of surface area covered by highlights play a profound role in judging how glossy a surface is (Beck & Prazdny, 1981; Di Cicco et al., 2019; Ferwerda et al., 2001; Marlow & Anderson, 2013; Marlow et al., 2012) and highlight features are also used to distinguish differences in gloss on a single surface (Wendt & Faul, 2019). We did not find any significant difference in matching ability between the two different albedos. Albedo is known to influence perceived gloss (Ferwerda et al., 2001; Hansmann-Roth & Mamassian, 2017; Hunter & Harold, 1987; Pellacini et al., 2000) however, the albedo did not vary between the target and the matching stimulus within a single trial and therefore the matching ability was unaffected by the albedo. We also found a difference in matching ability between the two different gloss levels. The matching error appears to be stronger for objects that were rendered with only a low gloss material. As previously mentioned, the transparent layer on top of the surface reduces contrast in the image. An object that is only slightly glossy and covered by a transparent layer might at some point show no visible highlights anymore or at least their contrast is highly reduced, in an extreme case the object will look like it is placed under diffuse lighting only. Therefore, it is reasonable that matching the material of a low gloss object is more difficult than for highly glossy objects.

#### Albedo Matching

Error bars for the albedo matching are larger than error bars for the gloss matching. Hence, the albedo matching of the object is far less consistent between observers. Lightness constancy has been found to be quite high for flat surface patches or Mondrian's (see, for example, Maertens & Shapley, 2013; Singh, 2004; Zeiner & Maertens, 2014). Lightness matching of more complex objects behind transparency seems to be far more complex. The environment map used to render the objects covers the entire surface. All transparent layers reduce the contrast, which results in consistent effects for the gloss matching. However, the different albedos of the transparent layers cause shifts to lower or higher luminance values. A dark transparent layer causes the images to appear entirely darker, whereas the light-colored transparent layer causes the entire image to appear brighter. We analyzed the percentage of pixels getting darker or lighter in the image after being placed behind the transparent layers. Almost all pixels (∼99%) are darker when the object is placed behind the darkest transparent layer, around 72%–76% of pixels are darker after the object is placed behind the second darkest layer and the majority of pixels are lighter after the object is placed behind the second lightest and lightest colored layers: 61%–77% for the second lightest layer and around 99% or the lightest colored layer. One might speculate that the overall darkening or lightening of the image is used as a cue/proxy for the observer to indicate the albedo of the material. Inferring the albedo behind a dark layer leads to the inference that the object has a lighter albedo and albedos behind a light-colored transparent layer must naturally be darker.

Todd et al. (2004) also examined lightness constancy on surfaces containing specular highlights on relatively simple, but not flat, objects. They presented smoothly curved surfaces to observers containing small rectangular patches of different albedos and illuminated by a point-light source provoking a specular highlight on the surface. Observers were instructed to compare the lightness of two rectangular albedo patches which occasionally contained the specular highlight. Their results showed that observers were able to discount the specular highlight and correctly estimate the albedo. However, their results also showed that when a deviation from lightness constancy occurred observers were biased towards judging the surface as lighter when the highlight was present compared to when the specular highlight was absent. This overestimation of the albedo was small expect for the darkest object. We did not find a significant interaction between albedo and the specular reflectance, but a trend was visible (*p* = .05). When just comparing the matching errors between the darker and the lighter objects used, it can be noticed that the matching error is slightly higher for the darker object than for the lighter object. This confirms the results by Todd et al. (2004) showing that specular highlights on dark surfaces induce a small overestimation of the albedo.

## Conclusions

In the current study, we investigated the role of transparency in gloss perception and observers’ degree of gloss constancy while seeing a glossy object through different transparent layers. In two separate experiments we showed that, overall, observers are quite precise when estimating gloss through transparency. However, observers were not able to fully account for the contributions that the transparent layer is inducing to the image. Estimating the reflectance properties of a surface requires the visual system to somehow decompose the image into its underlying causes. Our results for the first time show that the visual system is able to estimate gloss while objects are presented behind transparency and the induced histogram manipulations are taken into account.
